# Predicting Forest Tree Leaf Phenology Under Climate Change Using Satellite Monitoring and Population‐Based Genomic Trait Association

**DOI:** 10.1111/gcb.70484

**Published:** 2025-09-12

**Authors:** Markus Pfenninger, Liam Langan, Barbara Feldmeyer, Linda Eberhardt, Friederike Reuss, Janik Hoffmann, Barbara Fussi, Muhidin Seho, Karl‐Heinz Mellert, Thomas Hickler

**Affiliations:** ^1^ Senckenberg Biodiversity & Climate Research Centre Frankfurt/Main Germany; ^2^ IOME, J. Gutenberg University Mainz Germany; ^3^ Institute of Occupational, Social and Environmental Medicine J.W.Goethe University Frankfurt/Main Germany; ^4^ Bayerisches Amt für Waldgenetik, Bayerisches Staatsministerium für Ernährung, Landwirtschaft und Forsten Teisendorf Germany; ^5^ Department Biogeography J.W.Goethe University Frankfurt/Main Germany

**Keywords:** large scale phenotyping, leaf flushing, statistical prediction

## Abstract

Leaf phenology, a critical determinant of plant fitness and ecosystem function, is undergoing rapid shifts due to global climate change, yet its complex genetic and environmental drivers remain incompletely understood. Understanding the genetic basis of phenological adaptation is crucial for forecasting forest responses to a changing climate. Here, we integrate multi‐year satellite‐derived phenology from 46 *Fagus sylvatica* (European beech) populations across Germany with a population‐based genome‐wide association study to dissect the environmental and genetic drivers of leaf‐out day (LOD) and leaf shedding day (LSD). We show that environmental factors, particularly temperature forcing and water availability, are the primary drivers of LOD variation, while LSD is influenced by a more complex suite of climatic cues. Our genomic analysis identifies candidate genes associated with LOD and LSD, primarily linked to circadian rhythms and dormancy pathways, respectively. Furthermore, genomic prediction models incorporating these loci accurately reconstruct past phenological dynamics, providing a powerful framework to forecast forest vulnerability and adaptation to future climate change.

## Graphical abstract

We studied European beech trees to understand how the interaction between genetics and the environment, particularly climate change, influences the timing of leaf budding and leaf fall. We found that leaf budding in spring depends mainly on temperature and water, and we identified specific genes that are associated with both spring and autumn timing. With this knowledge, we were able to develop models that can better predict how forests will respond to future climate change.

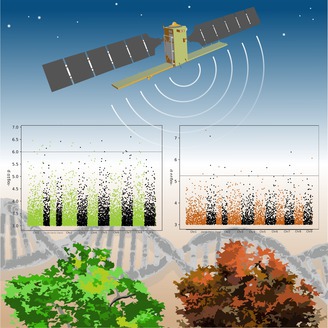

## 1 Introduction

Leaf phenology determines when and how long plants photosynthesize, directly influencing whether this activity aligns with favorable environmental conditions. In deciduous trees, leaf phenology links the vegetated land surface with atmospheric energy (e.g., albedo) and gas exchange (e.g., CO_2_ and water vapor), making it a key biological process with significant climate feedbacks (Richardson et al. 2013). For temperate deciduous trees, phenological timing is also a major fitness trait, balancing the benefits of a longer growing season with the risks of frost or drought (Alberto et al. 2013; Kramer et al. 2000; Li et al. 2023). Leaf phenology is triggered by environmental cues (Polgar and Primack 2011) but also has a genetic basis (Falusi and Calamassi 1996; Kramer 1995; Marchand et al. 2020; Robson et al. 2013; Vitasse et al. 2009), recently shown to be complex and polygenic (Satake et al. 2022). Like most quantitative traits (Walsh and Lynch 2018), it results from both environmental and genetic influences. Climate change is shifting environmental phenology cues in time and space, with major implications for forest ecosystems and climate dynamics (Peñuelas and Filella 2009; Richardson et al. 2013). With estimates of phenotypic reaction norms and genomic prediction scores accounting for heritable differences, forecasting phenotypes under novel conditions becomes feasible (Arnold et al. 2019). Accurate predictions therefore depend on disentangling environmental and genetic effects (Satake et al. 2022; Waldvogel et al. 2020). To improve understanding and forecasting of forest responses to climate change, it is crucial to integrate the environmental and genetic dimensions of phenological responses (Gray and Ewers 2021).

Until recently, large‐scale, long‐term phenotyping of phenological traits was logistically difficult and error‐prone (Gray and Ewers 2021). This is now changing with satellite‐based remote sensing, which provides spatially and temporally highly resolved phenotypic data suitable for genome‐wide association studies (GWAS) (Bian et al. 2022). Calibration with field data (Dronova and Taddeo 2022) has shown that Synthetic Aperture Radar is proven effective for capturing forest phenology with high accuracy (Proietti et al. 2020).

Leaf phenological variation in European beech ( *Fagus sylvatica* ) has been extensively studied (Čufar et al. 2008; Dittmar and Elling 2006; Dolschak et al. 2019; Wang et al. 2022); however, most efforts have focused on environmental correlates or candidate gene approaches (Krajmerová et al. 2017), leaving the genome‐wide basis of phenological adaptation at the landscape scale largely unexplored. Here, we integrate high‐resolution satellite‐based phenological monitoring with a novel population‐based genome‐wide association study (popGWAS) (Pfenninger 2025) to investigate the genetic and environmental drivers of spring and autumn leaf phenology. This approach, based on population allele frequencies, perfectly suits remote sensing data because it does not require individual phenotyping but exploits the mean trait differences among populations (Pfenninger 2025). Using a chromosome‐scale reference genome, multi‐year weather data (2015–2022), and phenological observations across 46 beech stands in Germany, we examine whether this integrated approach can address previous limitations and provide a foundation for large‐scale, long‐term forecasts of forest responses to climate change. European beech, a dominant forest species (Elsasser et al. 2021) supporting high biodiversity (Brunet et al. 2010; Dorow et al. 2010) and recognized by UNESCO for its primeval stands (Heim et al. 2018), serves as a model species to demonstrate how coupling large‐scale phenotyping with population genomics opens new pathways for forecasting ecosystem resilience under future climates.

## 2 Material and Methods

### 2.1 Study Populations

We sampled 46 mature beech‐dominated stands (> 70 years old, > 4.5 ha, ≥ 75% beech basal area) across Germany, emphasizing climatic and ecological diversity with a focus on the state of Hessen. Sites likely included both autochthonous and afforested origins, typical of German beech forests. To minimize sampling of close relatives, 48 canopy trees were collected per site, spaced ≥ 30 m apart.

### 2.2 Climatic Characterization

Long‐term climatic data (1970–2000) were extracted from WorldClim v2.1 (Fick and Hijmans 2017) at 30″ resolution for all sites and 180 random points across the species range, using DIVA‐GIS 7.5 (Hijmans et al. 2012) and species distribution shapefiles (Caudullo et al. 2017). We used all BioClim variables and summarized variation via Principal Component Analysis (PCA).

### 2.3 Remote Sensing‐Based Leaf Phenology Inference

Leaf phenology dates were inferred from Sentinel‐1 Synthetic Aperture Radar (SAR) data (VV and VH bands, 10 m resolution), comprising 9236 ortho‐corrected scenes (2015–2022) in Interferometric Wide Swath mode (Potin et al. 2016), accessed via Google Earth Engine. The backscatter of these microwave bands is, amongst other things, sensitive to the vegetation, in particular tiny structures, like leaves and twigs. Repeated recordings of the signal can therefore be used to monitor temporal changes in foliation. Microwaves can penetrate clouds and can therefore acquire information in all weather (Schlund 2025). The VV/VH backscatter ratio was calculated using geemap (Wu 2020) (Python 3.11.4), then squared to enhance the signal‐to‐noise ratio. Smoothed time series (LOESS, span = 0.5) were segmented into early and late season (cutoff = day 200). Change points representing maximum signal rate change were estimated using the changepoint package (Killick and Eckley 2014) (BinSeg, Q = 1; R v4.2.2). These changes correspond to the dates when about half of the leaves have emerged (LOD) and have fallen (LSD). The period between LOD and LSD was considered the maximum potential vegetation period. Ground‐truth leaf phenology data covering Germany from the German Weather Service (https://www.dwd.de/DE/leistungen/phaeno_sta/phaenosta.html) and the International Phenological Gardens (Renner and Chmielewski 2021) (station 189, Linden/Gießen) served as validation, using leaf phenology stage BBCH11 (first leaves completely unfolded) (Hack et al. 1992) (~LOD) and BBCH95 (50% of leaves fallen) (Hack et al. 1992) (~LSD) as comparison.

### 2.4 Environmental Drivers of Leaf Phenology

We assessed environmental influences on LOD and LSD using climate data from the German Weather Service (2015–2022, 1 km^2^ grid, https://cdc.dwd.de/portal/202209231028). Predictors for LOD included frost/ice days, temperature sums (Jan–Apr), precipitation (Mar–Apr), soil moisture (Mar–Apr), and latitude (photoperiod proxy). LSD predictors included temperature extremes, drought indices (de Martonne), soil moisture (May–Aug), and precipitation (May–Aug). Generalized Linear Models (GLMs) were fit for all parameter combinations; best‐fit models were selected using AIC. Site‐specific phenological offsets (residuals from the environmental models) were extracted using ANOVA and averaged over years (≥ 4 years per site required).

### 2.5 Pooled Sequencing and Variant Calling

From each individual, a 0.5 mm leaf disc (~50 mg) was collected, dried or frozen, and pooled by population. Samples were homogenized (Qiagen TissueLyser), and DNA extracted (Macherey‐Nagel NucleoMag Plant kit). Sequencing libraries were prepared and sequenced (150 bp paired‐end, ~35× coverage) by Novogene on an Illumina NovaSeq. Reads were trimmed (Trimmomatic v0.39), QC‐checked (FastQC v0.11.9), aligned to the beech reference genome (BWA mem v0.7.17), and processed (Samtools v1.10, Picard v2.20.8). PoPoolation2 v2.201 was used to remove indels, calculate allele frequencies, and compute FST values in non‐overlapping 1 kb windows (coverage range: 15–50×).

### 2.6 Population Structure and Leaf Phenology Associations

To test for confounding population structure, we performed PCA on allele frequencies, using one SNP per 50 kb with no missing data to reduce linkage. We then assessed correlations between the first five PCA axes and site‐specific leaf phenology offsets to verify independence between genetic structure and phenotypic variation.

### 2.7 SNP–Trait Association (popGWAS)

Using popGWAS (Pfenninger 2025), we regressed population mean offset in LOD and LSD against genome‐wide allele frequencies. Filtering retained SNPs with ≥ 15 mean coverage, AF variance ≥ 0.10, MAF ≥ 0.10, and ≤ 35% missing data. For each SNP, we computed linear models and extracted −log10 (*p*‐values). Outliers (top 0.01%) were identified and grouped into regions based on 1 kb linkage disequilibrium. The most differentiated SNP per region was retained. A scheme of the pipeline can be found in Figure S7.

### 2.8 Gene Annotation

Outlier SNPs were annotated using BEDtools intersect with the *F. sylvatica* v2 genome GFF (Mishra et al. 2021). We queried gene functions via UniProt and the ORKG Ask scholarly search and exploration system (ask.orkg.org) with prompts of the following structure “what is known about the role of [gene name] in determining [trait] day?”

### 2.9 Genomic Prediction

To predict unmeasured phenotypes, we built a statistical genomic prediction model based on significant SNPs. Missing values were imputed using coverage relaxation, linked SNPs (*r* > 0.7), or population means. Feature selection was performed via minimum entropy feature selection (MEFS) (Palamidessi and Romanelli 2018). The best predictive model was selected using repeated cross‐validation over varying feature counts (2 to *n*–1), implemented in scikit‐learn (v1.3.2) (Pedregosa et al. 2011). Predictive performance was tested on a hold‐out set of six populations using Pearson's *r*.

### 2.10 Candidate SNP Validation

LOD in 2024 was monitored in 14 juvenile beeches at site KST (April 1–29). Individual phenotypes were correlated with the mean number of LOD‐increasing alleles via BayesianFirstAid in R. LSD data were unavailable.

### 2.11 Postdicting Phenological Shifts

We evaluated whether adding genomic prediction scores (SGPS) improved LOD models via AIC. For 27 sites, environmental data since 1971 were used to postdict LOD shifts under observed environmental change using the best GLM + SGPS model.

## 3 Results

### 3.1 Climatic and Geographic Distribution of Sampling Sites

**FIGURE 1:**
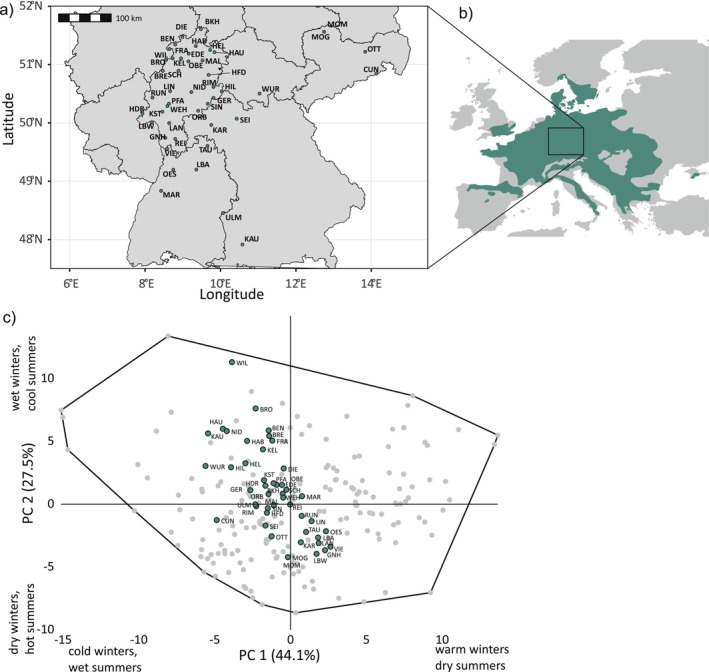
Geographic distribution and long‐term climate of *Fagus sylvatica* sampling sites. (a) Location of sampling sites. (b) Distribution range of *F. sylvatica* in Europe (dark green shading). (c) Principal Component Analysis (PCA) of long‐term climatic data (1960–1990) of the 46 sampling sites and 178 random points within the species range. The journal requires to state that map lines delineate study areas and do not necessarily depict accepted national boundaries.

The 46 sampling sites (Figure 1a) were selected from the core range of *F. sylvatica* (Figure 1b), spanning central climatic conditions of the species' distribution (Figure 1C). Climatic variation was primarily structured by two gradients: PC1 (44.1% variance) contrasted warm, dry summers with cold, wet winters, while PC2 (27.5%) ranged from wet winters and cool summers to dry winters and hot summers. Sites from the southeastern refugial region (Magri 2008; Tsipidou et al. 2021) overlapped climatically with much of the species' current range (Figure S1A,B).

### 3.2 Remote Sensing of Phenological Dates

Between 2015 and 2022, 62,570 remote sensing observations were collected—averaging 170 per site per year—allowing for near‐continuous phenological monitoring. We inferred 339 LOD and 340 leaf‐LSD estimates (~92% coverage; Figure S2). Five sites (WIL, WUR, GER, ORB, RIM) accounted for most missing data; WIL and ORB were excluded due to insufficient observations (< 4).

**FIGURE 2:**
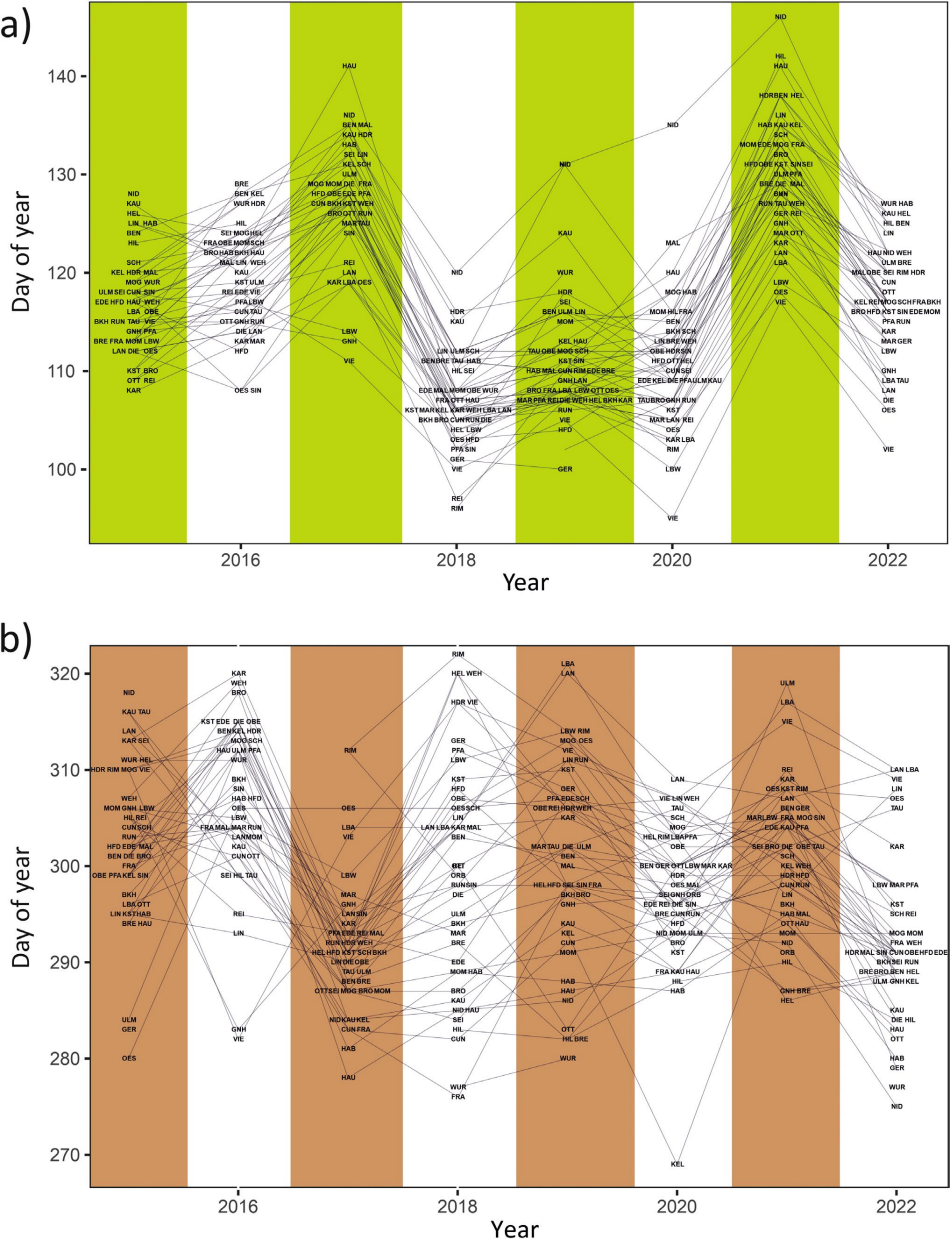
(a) Stand‐wide mean Leaves‐Out‐Day (LOD) inferred from Sentinel1 satellite data in the observation period 2015–2022. (b) Likewise for Leaves‐Shedding‐Day (LSD).

The mean LOD was 117.13 doy (s.d. 9.57, range 95–146), peaking in late April, and LSD averaged 298.99 doy (s.d. 10.12, range 269–322), peaking in late October. The potential vegetation period spanned an average of 181.72 days (s.d. 15.39, range 137–226 days). Both LOD and LSD varied significantly by year and site (Figure 2A,B). LOD was earliest in 2018 (mean 106.79 doy, s.d. 4.74) and latest in 2021 (mean 130.00 doy, s.d. 6.39). VIE had the earliest mean LOD (107.86, s.d. 8.59), and NID the latest (131.14, s.d. 8.9). For LSD, 2017 had the earliest onset (291.71, s.d. 7.39), and 2016 the latest (306.58, s.d. 8.86). BRE showed the earliest average LSD (289.57, s.d. 4.50), and LBA the latest (307.86, s.d. 8.71). The shortest maximum potential vegetation period averaged over all sites was inferred for the year 2017 (163.71 days, s.d. 11.80), the longest 1 year later in 2018 (192.59 days, s.d. 14.75; Figure S2A).

### 3.3 Validation of Remote Sensing Estimates

LOD estimates correlated well with publicly available Germany‐wide phenology data (*r* = 0.79, *p* = 0.01), lagging by 4.6 days on average (Figure S2A). A similar fit was seen between LOD from a phenological garden and a nearby site (LIN, *r* = 0.77, *p* = 0.026). For LSD, agreement with national data was moderate (*r* = 0.57, *p* = 0.12), with LSD leading by 14.5 days (Figure S2C). However, local LSD from the phenological garden did not correlate with the nearby site (*r* = 0.42, *p* = 0.29).

### 3.4 Environmental Drivers of LOD and LSD

To identify environmental drivers of phenological timing, we applied model selection based on previously proposed predictors (Essiamah and Eschrich 1986; Falusi and Calamassi 1990; J. Urban et al. 2013; Vitasse et al. 2009) (Table S1). For LOD, the best model included frost days, minimum temperatures in January to February, mean temperatures in March to April, and precipitation over those months. This model significantly outperformed alternatives (AIC = 2173; ∆AIC = 26.9; Table 1A), with all predictors having negative coefficients—indicating earlier leaf‐out under warmer, wetter conditions. Observed and modelled LOD were strongly correlated (*r* = 0.757, *p* < 2.2e‐16; Figure S4A).

**TABLE 1:** Results of model selection on phenological variation. (A) ANOVA table of best fit model and additional variance explained by systematic site effects on LOD. (B) Same for LSD.

(A) Effect	d.f.	SS	*p*	Coeffcient	Interpretation
Number of days with frost	1	6012	7.81e‐12	−0.35	More frost days trigger earlier leaf flushing
Sum of daily minimum temperatures Jan & Feb	1	1150	< 2e‐16	−0.62	Warmer winter triggers earlier flushing
Sum of daily mean temperature Mar & Apr	1	7400	< 2e‐16	−1.02	Warmer spring triggers earlier flushing
Mean soil moisture Mar & Apr	1	2385	4.39E‐07	−0.10	More precipitation in spring triggers earlier flushing
Residuals	328	13,142			
Site	43	4227	6.92e‐13		
Residuals	287	8916			
Total	332	30,090			

(B) Effect	d.f.	SS	*p*	Coefficient	Interpretation
Mean minimum temperature in Oct	1	107	0.00387	−1.15	
Number of hot days (> 30°C)	1	2428	7.87e‐12	0.71	Cold October nights induce earlier leaf‐fall
Mean de Martonne drought index May–Sep	1	56	4.45e‐06	−5.30	Hot days in summer prolong vegetation period
Mean soil moisture May–Sep	1	3241	1.99e‐09	0.58	Drought in summer induces earlier leaf‐fall
Residuals	311	26,374			Wetter soil in summer prolongs vegetation period
Site	45	7729	2.52e‐10		
Residuals	293	19,663			
Total	315	32,205			

LSD variation was less well captured. The best model (AIC = 2306.9; ∆AIC = 6.5) explained moderate variance (*r* = 0.432, *p* = 9.73e‐16; Figure S4B). Key predictors included mean minimum temperature in October, number of hot days (> 30°C), mean de Martonne drought index (May–September), and soil moisture over the same period (Table 1B). Cold autumn nights and dry summers accelerated senescence, while hot extremes and higher soil moisture tended to delay it.

### 3.5 Geographic Patterns in Phenology

LOD increased with both latitude and longitude, and was associated with colder, drier winters and cooler, wetter summers (Figure S3). LSD showed the opposite trend—decreasing with latitude and longitude, and with colder, wetter winters and cooler, wetter summers. Consequently, the length of the vegetation period varied widely: from just 159.3 days at NID to 210.3 days at RIM.

### 3.6 Site‐Specific Phenological Offsets

**FIGURE 3:**
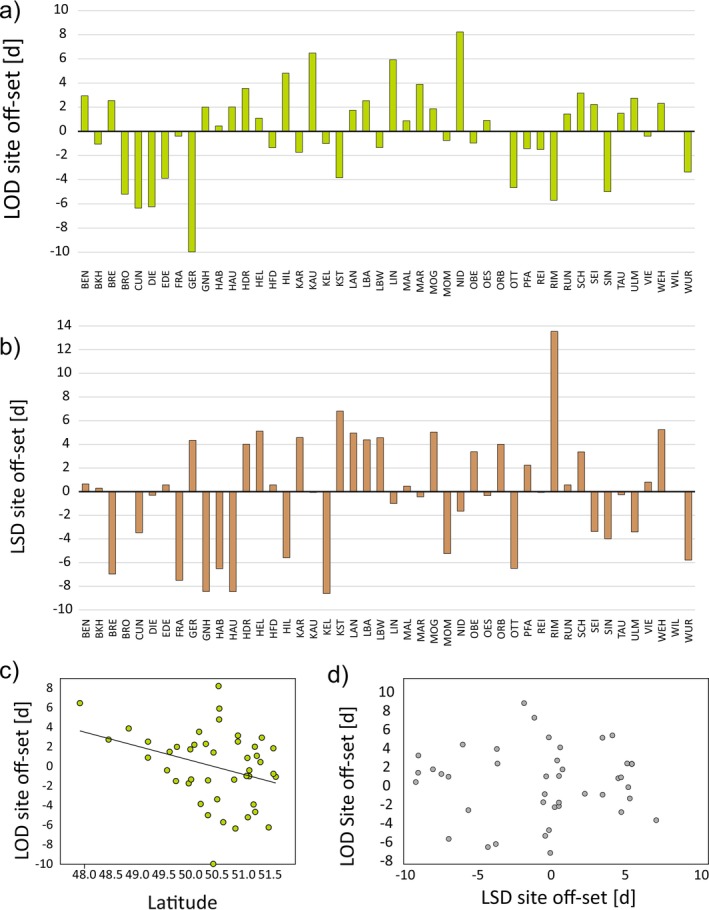
Site specific off‐sets in LOD and LSD. (a) Site specific off‐sets in LOD. (b) Site specific off‐sets in LSD. (c) Plot of the site specific LOD versus LSD effects. (d) Plot of LOD site offset against latitude.

On average, sites deviated from predicted LOD values by 2.98 days (s.d. = 2.24). The spread between the earliest (GER, −9.97 days) and latest (NID, +8.23 d) site offset was 18.2 days (Figure 3a). LSD offsets ranged even more: from RIM (+13.54 d) to KEL (−8.60 d), a span of over 22 days. The mean offset for LSD was 3.72 days (s.d. = 3.03; Figure 3b).

LOD offsets weakly correlated with latitude (*r* = −0.327, *p* = 0.030), with northern sites flushing earlier than expected (Figure 3c). LSD offsets showed weak positive correlations with long‐term winter/early‐spring maximum temperatures (*r* ≈0.31–0.32, *p* = 0.030–0.048), suggesting later leaf‐fall under warmer conditions. Notably, LOD and LSD offsets were uncorrelated (*r* = 0.035, *p* = 0.82; Figure 3d).

### 3.7 popGWAS Results

None of the first five PCA axes (explaining together 17.6% of total allele frequency variation as measurement of population structure) correlated with site‐specific LOD or LSD offsets (Table S2). Combined with low population differentiation (mean F_ST_ = 0.062, s.d. = 0.002), this supported the validity of applying popGWAS (Pfenninger 2025).

**FIGURE 4:**
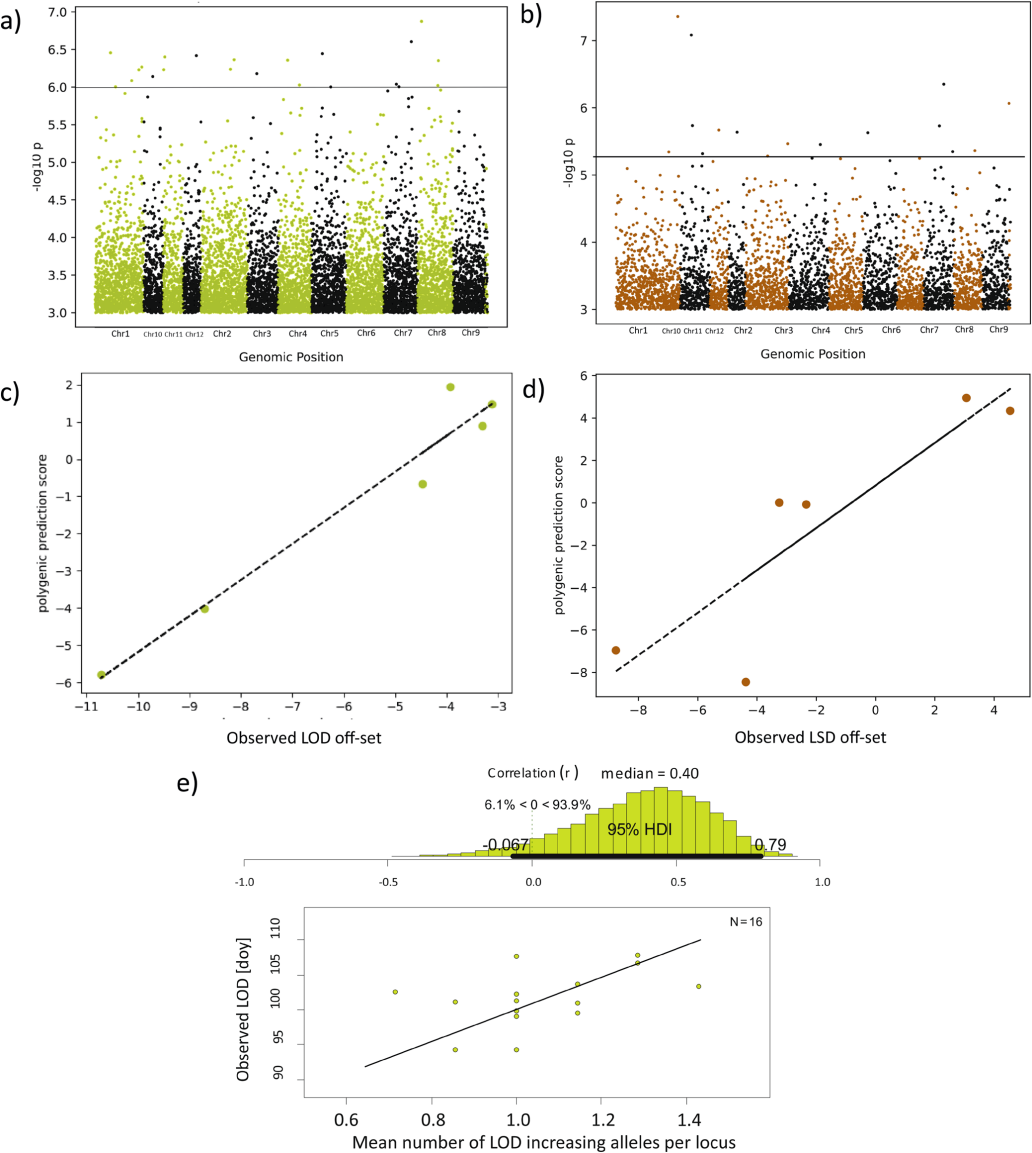
Manhattan plots of popGWAS results and validation of genomic prediction. Shown are the −log10 *p*‐values above 3.0 along the chromosomes of *F. sylvatica* . (a) Plot for LOD. The horizontal line marks the chosen cut‐off above which SNPs were considered as candidates. (b) The same for LSD. (c) Plot of observed LOD off‐set scores against the polygenic prediction score derived from hold out populations. (d) The same for LSD. (e) Functional validation. Bayesian correlation analysis of the mean number of LOD‐increasing alleles at predictive loci in 16 individually sequenced trees against their observed LOD date in 2024.

At a 0.9999 threshold (−log_10_ *p* > 6.0), 22 SNPs were associated with LOD offsets (Figure 4A), spread across all except one chromosome (Chromosome 9, Table 2A). Ten fell within genes, four within 2 kb of genes, and eight in intergenic regions. Among genic SNPs: 3 were intronic, 2 synonymous, and 5 non‐synonymous with potentially large protein effects (Table S2A). Of the 14 genes associated with these SNPs, 8 were linked to circadian regulation or leaf flushing (Table 2B), while 6 involved retrotransposon elements, or were uncharacterized.

**TABLE 2:** Annotation of outlier SNPs. (A) Outlier SNPs for LOD, (B) outlier SNPs for LSD.

(A)							
CHR	POS	Location	Gene ID	Uniprot ID	Gene name	Potential function in LOD	Citation
Bhaga_1	29,694,923	Intergenic	—	—	—	—	—
Bhaga_1	36,902,340	In gene	Bhaga_1.g3485	RVW73694.1	Retrovirus‐related Pol polyprotein from transposon 297	Unclear	—
Bhaga_1	56,460,105	In gene	Bhaga_1.g5529	XP_023872109.1	Probable ribose‐5‐phosphate isomerase 3	Diurnal variation of isoprene synthase (ISPS) activity and gene expression in poplar trees, potentially affecting leaf out day through the involvement of this enzyme in the pentose phosphate pathway	Loivamäki et al. (2007)
Bhaga_1	66,760,356	In gene	Bhaga_1.g6656	XP_023921151.1	F‐box/kelch‐repeat protein At3g06240‐like	Significant role in the regulation of plant circadian clocks and flowering time by sensing dusk	Feke et al. (2021)
Bhaga_1	71,383,965	In gene	Bhaga_1.g7194	RVX01392.1	Transposon TX1 uncharacterized 149 kDa protein	Unclear	—
Bhaga_10	18,380,215	In gene	Bhaga_10.g2246	XP_018820584.1	Histidine—tRNA ligase	The expression and regulation of a histidine—tRNA ligase gene might influence the H2B monoubiquitination status, which in turn could affect the timing of gene expression, including leaf out day	Baerenfaller et al. (2016); Skaf (2012)
Bhaga_11	390,980	In gene	Bhaga_11.g48	XP_023899280.1	Wall‐associated receptor kinase 2‐like	Potentially impact stomatal function and the circadian rhythm of stomatal opening both of which influence leaf out day.	Lebaudy et al. (2008)
Bhaga_11	2,383,739	In gene	Bhaga_11.g261	XP_023870530.1	E3 SUMO‐protein ligase KIAA1586‐like	Role in regulating key clock properties in the model plant *Arabidopsis*	Hansen et al. (2017)
Bhaga_12	20,600,635	±2 kb	Bhaga_12.g2433	XP_018836710	Receptor protein kinase‐like protein ZAR1	Unclear	—
Bhaga_2	38,116,095	In gene	Bhaga_2.g4150	RVW75728.1	Retrovirus‐related Pol polyprotein from transposon RE1	Unclear	—
Bhaga_2	41,013,910	Intergenic	—	—	—	—	—
Bhaga_3	15,569,811	Intergenic	—	—	—	—	—
Bhaga_4	13,462,947	In gene	Bhaga_4.g1582	XP_023913658.1	Probable xyloglucan endotransglucosylase/hydrolase protein 30	Might impact leaf out day by influencing cell wall modifications	Becnel (2006)
Bhaga_4	31,490,757	Intergenic	—	—	—	—	—
Bhaga_5	13,093,530	±2 kb	Bhaga_5.g1475	RVW68719.1	Transposon Ty3‐I Gag‐Pol polyprotein	Unclear	—
Bhaga_5	23,175,138	±2 kb	Bhaga_5.g2628	XP_030966320.1	Transcription termination factor MTERF9	Unclear	—
Bhaga_7	20,188,845	±2 kb	Bhaga_7.g2359	XP_030929277.1	Putative disease resistance protein RGA3	Unclear	—
Bhaga_7	22,393,706	In gene	Bhaga_7.g2591	XP_030924992.1	Uncharacterized protein	Unclear	—
Bhaga_7	35,653,971	Intergenic	—	—	—	—	—
Bhaga_8	8,482,558	Intergenic	—	—	—	—	—
Bhaga_8	25,606,811	In gene	Bhaga_8.g3097	—	—	—	—
Bhaga_8	25,807,997	Intergenic	—	—	—	—	—

(B)							
CHR	POS	Location	Gene ID	Uniprot ID	Gene name	Potential function LOD	Citation
Bhaga_1	12,763,752	Intergenic	—	—	—	—	—
Bhaga_1	58,851,447	Intergenic	—	—	—	—	—
Bhaga_1	70,080,082	±2 kb	Bhaga_1.g7039	XP_023926929.1	40S ribosomal protein S2‐3‐like	Might be involved in leaf fall day through its role in senescence and plant tolerance to chilling stress	Ekramoddoullah et al. (1995); Jehanzeb et al. (2017)
Bhaga_10	14,094,514	In gene	Bhaga_10.g1727	RVW63395.1	Retrovirus‐related Pol polyprotein from transposon TNT 1–94	Unclear	—
Bhaga_10	15,451,591	In gene	Bhaga_10.g1898	XP_030924638.1	Uncharacterized protein LOC115951608	Unclear	—
Bhaga_10	28,050,045	Intergenic	—	—	—	—	—
Bhaga_11	4,076,294	±2 kb	Bhaga_11.g459	ACG58885.1	Cinnamyl alcohol dehydrogenase	Unclear	—
Bhaga_11	14,934,036	In gene	Bhaga_11.g1682	PNY15174.1	Ribonuclease H	Might be involved in regulating fall dormancy (FD) in alfalfa through the expression of specific genes such as ILR1‐like 1, PYL8, and monogalactosyldiacylglycerol synthase‐3	Du et al. (2017); Zhang et al. (2015)
Bhaga_12	13,977,597	±2 kb	Bhaga_12.g1708	PNY17872.1	Putative gag‐pol polyprotein	Unclear	—
Bhaga_2	28,990,891	Intergenic	—	—	—	—	—
Bhaga_2	53,382,765	In gene	Bhaga_2.g5932	XP_018824994.1	Exopolygalacturonase‐like	Might play a role in the leaf fall day by regulating fall dormancy (FD)	Du et al. (2017); Zhang et al. (2015)
Bhaga_3	24,739,929	Intergenic	—	—	—	—	—
Bhaga_3	35,852,818	±2 kb	Bhaga_3.g4054	XP_023889866.1	Exosome complex exonuclease RRP44 homolog A‐like	UNCLEAR	—
Bhaga_4	17,419,372	In gene	Bhaga_4.g2027	RVW68719.1	Transposon Ty3‐I Gag‐Pol polyprotein‐	Unclear	—
Bhaga_5	10,441,249	In gene	Bhaga_5.g1169	XP_023876123.1	ABC transporter A family member 2‐like	ABC transporter A family member 2 genes, such as those encoded by PYL8 in alfalfa, have been implicated in regulating fall dormancy (FD) in plants	Du et al. (2017); Zhang et al. (2015)
Bhaga_5	36,823,695	In gene	Bhaga_5.g4208	XP_030929469.1	G‐type lectin S‐receptor‐like serine/threonine‐protein kinase At4g27290 isoform X2	Unclear	—
Bhaga_6	36,437,472	±2 kb	Bhaga_6.g4081	XP_010446646.1	Egg cell‐secreted protein 1.1‐like	Might be involved in the regulation of fall dormancy by programmed cell death	Stahl et al. (2023)
Bhaga_7	24,473,847	In gene	Bhaga_7.g2826	XP_030971281.1	Probable LRR receptor‐like serine/threonine‐protein kinase At1g56140	Might play a role in the regulation of leaf fall day through the activation of mitogen‐activated protein kinases (MAPKs)	Chandra et al. (2016); Wang et al. (2023)
Bhaga_7	29,440,369	In gene	Bhaga_7.g3376	XP_030970327.1	Dynamin‐related protein 4C‐like	Might be involved in leaf fall day through its role in dormancy, starch degradation and cold acclimation	Du et al. (2017)
Bhaga_7	40,035,298	±2 kb	Bhaga_7.g4650	XP_030973049.1	CASP‐like protein 2B1	Might be involved in regulating leaf fall day through its role in frost hardiness or fall dormancy	Du et al. (2017); Ekramoddoullah et al. (1995)
Bhaga_8	30,834,279	In gene	Bhaga_8.g3674	XP_027190985.1	Protein NYNRIN‐like	Might be related to leaf fall day based on findings in poplar trees. In poplar species, a bark storage protein (BSP) accumulates during the fall and winter	Du et al. (2017); Thomas et al. (2002)
Bhaga_Unplaced_1597	178,270	±2 kb	Bhaga_Unplaced_1597.g119	NA		Unclear	

For LSD, 22 candidate SNPs surpassed the 0.9999 threshold (−log_10_ *p* > 5.2, Figure 4b). Of these, 10 were genic, 7 near genes, and 5 intergenic (Table 2B). Six were intronic, one synonymous, and three non‐synonymous (Table S2B). Nine associated genes had plausible or known roles in leaf senescence. Transposon‐related genes accounted for 4 (LOD) and 3 (LSD) SNPs. For both traits, SNPs on the same chromosome were well separated (≥ 1.7 Mb; mean = 14 Mb).

### 3.8 Genomic Prediction and Functional Validation

LOD phenotypes were best predicted by Minimum Entropy Feature Selection using allele frequencies at 10 selected candidate SNPs (model score > 0.99), achieving 0.97 accuracy in six hold‐out populations (Figure 4c). Similarly, 10 SNPs predicted LSD with a score of 0.98 and a validation accuracy of 0.88 (Figure 4d).

Of these 10 predictive loci, 7 were polymorphic in 16 independently whole‐genome sequenced individuals with available LOD data for the year 2024. Bayesian correlation analysis revealed a 93.9% posterior probability for a positive link between the mean number of LOD‐increasing alleles per locus and the observed LOD phenotype, with a moderate median correlation (*r* = 0.40; 95% HDI: −0.067 to 0.79; Figure 4e).

### 3.9 Modeling Historical Phenological Change

**FIGURE 5:**
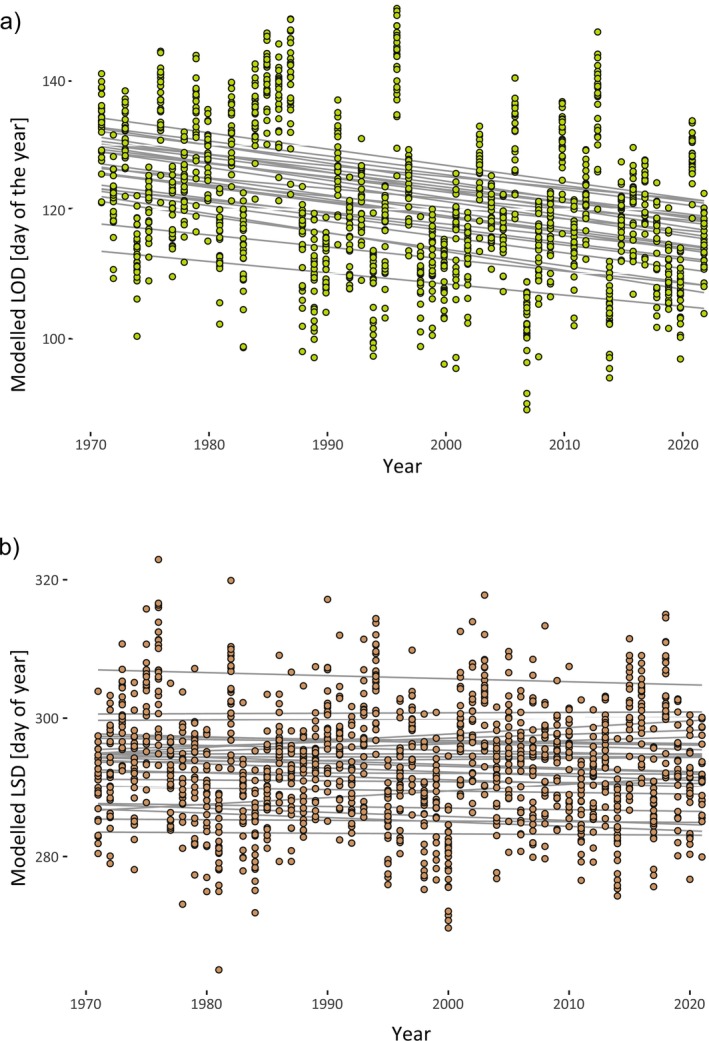
Postdictive modelling of phenological dates for the period 1971–2022. (a) LOD modelled for a set of 27 sites. (b) LSD modelled for the same sites. The grey lines correspond to the slopes of a linear model fitted for each site.

Adding genomic prediction scores to environmental data significantly improved predictive models for both LOD (∆AIC = 224.7) and LSD (∆AIC = 138.7), increasing correlations with observed values to *r* = 0.835 and *r* = 0.446, respectively. Using these enhanced models to reconstruct phenology between 1971 and 2022, LOD shifted significantly earlier (slope = −0.16, *p* = 1.28e–27), advancing by ~1.6 days per decade, or ~8 days total since the 1970s (Figure 5a). While a linear trend described this shift reasonably, a sigmoidal Hill function provided a much better fit (∆AIC = 416.8, Figure S5A). This model identified an abrupt change around 1988, with mean LOD dropping from day 127.4 to 117.6, coinciding with a sharp spring warming (Figure S5B). LSD, in contrast, remained stable near the end of October throughout the period (slope = 0.009, *p* = 0.545; Figure 5b). As a result, the potential vegetation period expanded significantly (slope = −0.22, *p* = 3.4e–13, Figure S6B), increasing from ~167 to ~178 days over five decades.

## 4 Discussion

By integrating satellite‐based phenotyping with a novel GWAS framework, we assessed phenological variation across *F. sylvatica* stands with unprecedented spatial and temporal resolution. This allowed us to partition trait variance into environmental and genetic components, identify candidate loci underlying heritable variation, and build accurate genomic prediction models across the species' core range.

Satellite remote sensing proved highly effective for large‐scale phenotyping, enabling consistent leaf status tracking over multiple years—at a resolution that would have required significant logistical and financial investment using field‐based methods. Although our primary goal was to capture a reliable, objective signal of canopy‐level phenological transitions, prior work (Proietti et al. 2020) confirms that the strongest change point aligns well with internationally recognized phenological stages. The strong agreement with publicly available phenology data (despite a consistent time lag) supports the reliability of our remote‐sensed estimates. Still, limitations exist. Dense undergrowth of beech—particularly where it retains winter foliage—can obscure canopy signals, occasionally preventing accurate LOD or LSD detection. Fortunately, such cases were rare and did not materially impact our analyses.

Both leaf emergence (LOD) and leaf shedding (LSD)—and thus the total growing season—were primarily driven by environmental variation. As expected from previous studies (Essiamah and Eschrich 1986; Falusi and Calamassi 1990; Urban et al. 2013; Vitasse et al. 2009), early‐spring temperature forcing and water availability best explained spatial and temporal variation in LOD, accounting for ~56% of its variance. In contrast, LSD was shaped by competing effects: cold October nights and dry summers accelerated leaf fall, while very hot days and high soil moisture tended to delay it. Although prior work (Lukasová et al. 2020) reported heat as a trigger for early leaf coloring, this may still align with our findings—damaged leaves might senesce earlier but remain attached longer. However, environmental predictors were less effective for LSD. This likely reflects local, stochastic events—like windstorms or heavy rain—that can influence the exact timing of leaf fall but are not captured in large‐scale climate data (Mariën et al. 2019).

LOD and LSD both showed opposing trends with geographical and climatic gradients, suggesting strong environmental control over the vegetation period, which in turn likely shapes beech distribution—particularly at northern and eastern range limits. This complements previous work highlighting drought susceptibility as a key constraint in the east (Roibu et al. 2017, 2022). Postdictive modeling supported this interpretation: climate change has already lengthened the growing season at the study sites, mostly through earlier LOD rather than delayed LSD, which is contrary to findings in other studies (Garate‐Escamilla et al. 2020). The observed advance of ~8 days since the 1970s was not linear. Instead, a sigmoidal model fit the data better, pointing to an abrupt shift in the late 1980s, coinciding with a well‐documented surge in spring temperatures (Menzel et al. 2006).

Both leaf emergence (LOD) and leaf shedding (LSD) showed signs of local adaptation, with likely heritable components varying systematically across space and time. For LOD, site‐specific offsets not explained by environmental variables—that is, the variance where genetic differentiation may reside (Visscher et al. 2008) —accounted for ~14% of the total variation. These offsets negatively correlated with latitude, suggesting that northern stands initiate leaf‐out earlier than expected based on climate alone, likely compensating for photoperiod constraints via local genetic tuning (Garate‐Escamilla et al. 2020). This supports previous findings of local adaptation in leaf emergence timing (Lazic et al. 2024; Meger et al. 2021), and implicates photoperiod perception as a driver (Zohner and Renner 2015).

Site‐specific offsets in LSD were even more substantial, explaining ~24% of variance. Stands with generally warmer winters—where frost risk is lower—tended to retain their leaves longer, again hinting at local genetic adaptation. Though this association was relatively weak, it is the first evidence of such a pattern in European beech. Crucially for the GWAS approach, the presumed genetic components of LOD and LSD were entirely uncorrelated, implying distinct genetic architectures for these traits (Cheverud 1988). This independence means natural or artificial selection could potentially act on leaf‐out and leaf‐drop timing separately (Svensson et al. 2021). Moreover, predicting the effect of climate change on the length of the growing season requires considering these traits separately.

This study represents the first empirical application of the popGWAS approach (Pfenninger 2025). Unlike traditional GWAS, which links individual genotypes to phenotypes, popGWAS uses genome‐wide allele frequencies to explain population‐level phenotypic variation, offering greater statistical power with the same sequencing effort. Simulations show that popGWAS reliably detects true positive loci for polygenic traits when at least ~36 populations are analyzed and a few dozen top outlier candidates are considered. We analyzed 46 populations, well above the recommended minimum, and selected 22 top outlier regions per trait as a conservative approach for *F. sylvatica* 's 0.5 Gb genome (Pfenninger 2025). A key requirement for successful application is weak population structure unrelated to the traits under study, which was met, as evidenced by the low F_ST_ (0.062) and lack of correlation between PCA axes and the genetic components of LOD and LSD.

Many identified outlier loci were located in or near genes known or plausibly related to the traits, making the associations highly unlikely to be due to chance. For LOD, eight genes were linked to the circadian clock or leaf flushing, with similar roles in regulating leaf emergence in tree species like poplar (Edwards et al. 2018; Ibáñez et al. 2010). Circadian clock components were shown to be crucial in regulating various growth activities in for example, poplar trees, including leaf emergence, by modulating gene expression and physiological processes by synchronizing these processes with environmental cues such as light and temperature (Wenden et al. 2012). For LSD, nine genes were previously associated with fall dormancy or leaf fall (Du et al. 2017; Zhang et al. 2015). A notable proportion of candidate SNPs were found in transposon genes, where polymorphisms are known to influence gene expression (Linker and Hedges 2013). However, validation and fine mapping of these loci are necessary to confirm their functional roles (Cano‐Gamez and Trynka 2020). Notably, a positive correlation between the mean number of LOD‐increasing alleles and observed LOD in an independent dataset of 16 sequenced beech trees suggests a significant functional role of these SNPs.

We successfully developed accurate statistical models to predict the site‐specific genetic component of phenotypes. Prediction accuracy for hold‐out populations was high—0.97 for LOD and 0.88 for LSD—despite LSD's greater environmental sensitivity. This level of success is notable given the modest performance of genomic prediction in other contexts (Visscher et al. 2017; Wray et al. 2019). For a correct evaluation, it is essential to distinguish the dual aims of GWAS: identifying causative loci and predicting outcomes. While related, explanation and prediction are distinct. A model can predict well without fully capturing underlying mechanisms, as long as key features correlate strongly with the outcome (Shmueli 2010). Although including more functional loci can enhance predictive accuracy (Pfenninger 2025), it is not necessary to include all or even most trait‐related loci. Thus, our model should not be interpreted as a comprehensive functional (quantitative) genetic model. Accurate predictions of phenotypic means can emerge when allele frequency differences at just a few loci follow a linear pattern, while other loci behave nearly neutrally. The imperfect fit of outlier loci to linear models reflects the redundancy typical of polygenic traits (Barghi et al. 2020). Still, if key loci are shared across populations—via shared ancestry or gene flow—accurate predictions remain feasible. These loci are typically of intermediate frequency, as rare alleles contribute little to variation in population means, regardless of effect size (Pfenninger 2025). Conversely, individual phenotypes may be shaped by rare alleles of large effect (Pickrell et al. 2016; Sinclair‐Waters et al. 2020). Thus, loci important for mean differences among populations may offer limited predictive power at the individual level. Predicting individual phenotypes likely requires a broader set of loci.

Contrary to its application in medicine or selective breeding (Wray et al. 2019), however, accurate prediction of population responses is in many instances of the current biodiversity crisis probably more important than the prediction of individual phenotypes (Urban et al. 2024; Waldvogel et al. 2020). Within these limits, our approach yielded highly accurate predictions. While not functionally comprehensive (de Los Campos et al. 2018), these prediction scores reliably correlate with trait means and may extend across much of the central *F. sylvatica* range (Lazic et al. 2024; Magri 2008; Postolache et al. 2021), given its low genetic differentiation. Such scores could support identifying populations with adaptive potential under future climates.

The polygenic nature of both traits, the pronounced genetic offsets among populations together with the low population differentiation and high effective population size suggests that there is ample opportunity for natural selection to fine‐tune phenological dates in European beech according to the changing conditions also in the future. However, this presupposes that forest management is carried out in a way that maintains genetic diversity and allows natural selection to take place (Gömöry et al. 2020; Ratnam et al. 2014).

Our study highlights the power of combining satellite remote sensing with a novel population‐based GWAS to assess phenological variation in *F. sylvatica* at an unprecedented scale. Remote sensing enabled consistent, large‐scale phenotyping across years, uncovering spatial and temporal patterns in leaf‐out and senescence that traditional methods would struggle to capture. By separating genetic from environmental influences, we provide strong evidence for local adaptation and identify candidate genomic regions involved in phenological regulation.

Our predictive modeling further demonstrates the potential of integrating genomic and remote sensing data to forecast population‐level phenotypic responses—critical for conservation and forest management under climate change. While functional validation of key loci and refinement of remote sensing techniques remain important next steps, our findings underscore the value of linking high‐throughput ecological monitoring with genomic tools to better predict biodiversity responses and inform adaptation strategies for long‐lived tree species.

## Author Contributions

**Markus Pfenninger:** conceptualization, data curation, formal analysis, funding acquisition, methodology, project administration, writing – original draft. **Liam Langan:** conceptualization, writing – review and editing. **Barbara Feldmeyer:** data curation, formal analysis, writing – review and editing. **Linda Eberhardt:** data curation, writing – review and editing. **Friederike Reuss:** data curation, funding acquisition, writing – review and editing. **Janik Hoffmann:** data curation, formal analysis, writing – review and editing. **Barbara Fussi:** data curation, writing – review and editing. **Muhidin Seho:** conceptualization, writing – review and editing. **Karl‐Heinz Mellert:** conceptualization, writing – review and editing. **Thomas Hickler:** conceptualization, writing – review and editing.

## Conflicts of Interest

The authors declare no conflicts of interest.

## Supporting information


**Data S1:** gcb70484‐sup‐0001‐DataS1.docx.

## Data Availability

The raw data of all samples sequenced can be found at ENA (study numbers: PRJEB64934 and PRJEB60881). Other data can be found at Zenodo https://doi.org/10.5281/zenodo.15401304. Scripts can be found at Zenodo https://doi.org/10.5281/zenodo.15341395. WorldClim 2.1 data is deposited at https://doi.org/10.5281/zenodo.8319614. Current Sentinel–1 Synthetic Aperture Radar (SAR) data are available through the Google Earth Engine cloud–processing platform (https://developers.google.com/earth‐engine/datasets/catalog/COPERNICUS_S1_GRD?hl=de). Phenological data was obtained from German Weather Service (DWD) via their service platform (https://www.dwd.de/DE/leistungen/phaeno_sta/phaenosta.html). Historical weather data was downloaded from the DWD portal (https://cdc.dwd.de/portal/202209231028).
